# The complete chloroplast genome of *Pollia japonica* (Commelinaceae) from Southeast China

**DOI:** 10.1080/23802359.2021.1911717

**Published:** 2021-04-21

**Authors:** Ye Gu, Qing Ma

**Affiliations:** a Department of Gastroenterology, Key Laboratory of Clinical Cancer Pharmacology and Toxicology Research of Zhejiang Province, Affiliated Hangzhou First People's Hospital, Zhejiang University School of Medicine, Hangzhou, Zhejiang, P.R.China; b College of Biological and Environmental Engineering, Zhejiang Shuren University, Hangzhou, P.R.China

**Keywords:** Chloroplast genome, commelinales, phylogeny, Pollia japonica

## Abstract

The complete chloroplast genome of *Pollia japonica*, a medicinal herb native to East Asia was characterized. The size of the chloroplast genome is 165,076 bp in length with a large single copy (LSC) of 90,722 bp, a small single copy (SSC) of 19,146 bp, and a pair of inverted repeats of 27,604 bp. The chloroplast genome encodes a set of 131 genes, including 85 protein-coding, 38 tRNAs, and 8 rRNAs. Phylogenetic analysis based on complete chloroplast genomes validated the phylogenetic position of *P. japonica* and showed that the six species from Commelinales were fully resolved in a monophyletic clade sister to the Zingiberales. Species from the Zingiberales and Commelinales formed a molophyletic group sister to the Poales. The chloroplast genome of *P. japonica* provides an important resource for further study of molecular evolution in the Commelinaceae.

*Pollia japonica* is a perennial herb classified into the genus *Pollia* in the Commelinaceae family. Naturally distributed in East Asia, *P. japonica* has long been recognized as a medicinal plant (Zeng et al. 2017). The bright green leaves of *P. japonica* make it an excellent foliage plant with ornamental value. It has been reported before that species from the Commelinales demonstrate a typical system of morphologically convergent evolution (Givnish et al. 1999). Therefore, in this study, we report the complete chloroplast genome of *P. japonica*, which mays provide genetic resources for future phylogenetic studies on the Commelinales.

The specimen of *Pollia japonica* was collected from Lingfeng Hill in Hangzhou Botanical Garden (30.26 N, 120.12 E), Zhejiang Province, China. Total genomic DNA of *Pollia japonica* was extracted from fresh young leaf using the CTAB method (Doyle and Doyle 1987). The voucher specimen (No. 2020606) was deposited in Zhejiang Shuren University (Contact: Qing Ma, maqing90@live.cn). The chloroplast genome was sequenced using the Illumina Hiseq Platform (Illumina, San Diego, CA) at BGI (Shenzhen, China). Genome assembly and annotation were performed using *Commelina communis* (GenBank accession number: MK863371) as the reference genome with GetOrganelle (Jin et al. 2020) and Geneious R8 (Biomatters Ltd, Auckland, New Zealand), respectively. The start and stop codon positions as well as boundaries between exons and introns were manually checked and corrected. The annotated complete chloroplast genome of *Pollia japonica* was deposited to GeneBank under the accession number MW145132.

The chloroplast genome of *Pollia japonica* has a typical quadripartite structure consisting of a pair of inverted repeats (IRa and IRb: 27,604 bp), a small single copy (SSC) region (19,146 bp), and a large single copy (LSC) region (90,722 bp). The total length of the chloroplast genome was 165,076 bp and encodes 131 genes including 85 protein-coding genes, 38 tRNA genes, and 8 rRNA genes. The GC content of the whole genome is 35.8%. The chloroplast genome structure and gene locations of *P. japonica* are highly similar to the previously reported genomes of *C. communis* (MK863371), *Pontederia cordata* (NC_044968) and *Hanguana malayana* (NC_029962) from the Commelinales (Barrett et al. 2013; Cui and Liang 2019; Ma and Liang 2019).

Phylogenomic analysis based on the complete chloroplast genome was conducted using the maximum likelihood (ML) optimality criterion implemented on the CIPRES Science Gateway V.3.3 (Miller et al. 2010; Stamatakis 2014) using the GTR + I + G nucleotide substitution model and 1000 bootstrap replicates. A total of 28 species from Commelinales, Zingiberales, Poales, and Arecales were included in the matrix. Two species from Orchidaceae, Asparagales (*Dendrobium bellatulum* and *Dendrobium candidum*) were designated as outgroups. The chloroplast genome sequences of all the species used for the phylogenomic analyses were downloaded from GenBank and aligned with MAFFT version 7.0 using the default settings (Katoh et al. 2019).

The final phylogenetic tree strongly supported a close relationship between *P. japonica* and five species classied in the Commelinales. In addition, *P. japonica* and *C. communis* from the Commelinaceae were fully resolved on a branch with 100% support (Figure 1). All the species from Commelinales formed a monophyletic group sister to the Zingiberales. These evolutionary findings between Commelinales, Zingiberales, Poales, and Arecales are consistent with the results from previous phylogenetic studies (Figure 1; Givnish et al. 1999; Barrett et al. 2013; Grahan et al. 2006). This investigation provides useful genetic resources for further robust study on the phylogeny of Commelinales.

**Figure 1.**
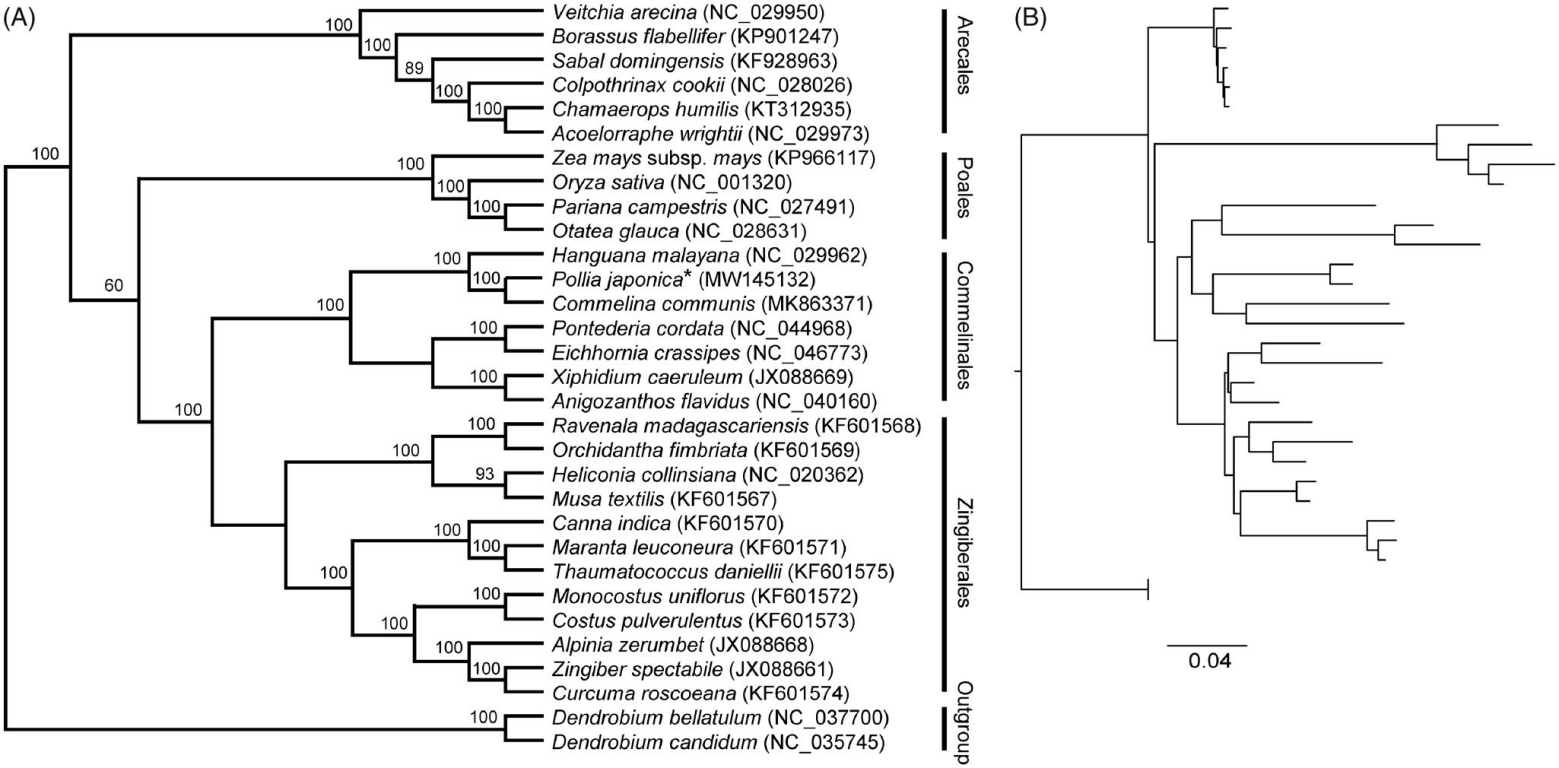
Phylogenetic tree reconstruction of *Pollia japonica* (marked with star) and other species from Commelinales, Zingiberales, Poales, and Arecales using maximum likelihood (ML) analysis based on whole chloroplast genome sequences. The tree topology (A) and relative branch lengths (B) are indicated. Numbers above each branch indicate the bootstrap values based on 1000 replicates obtained from ML analysis. GenBank accession numbers of all the chloroplast genome are shown in the brackets.

## Data Availability

The genome sequence data that support the findings of this study are openly available in GenBank of NCBI at (https://www.ncbi.nlm.nih.gov/) under the accession no. MW145132. The associated BioProject, Bio-Sample, and SRA numbers are PRJNA668921, SAMN16427400, and SRR13711041.
